# Involment of RAS/ERK1/2 signaling and MEF2C in miR-155-3p inhibition-triggered cardiomyocyte differentiation of embryonic stem cell

**DOI:** 10.18632/oncotarget.21218

**Published:** 2017-09-23

**Authors:** Xiang Ling, Dongbo Yao, Lumei Kang, Jing Zhou, Ying Zhou, Hui Dong, Keping Zhang, Lei Zhang, Hongping Chen

**Affiliations:** ^1^ Department of Thoracic Surgery, The First Affiliated Hospital of Nanchang University, Nanchang, Jiangxi 330006, China; ^2^ Department of Histology and Embryology, Medical College, Nanchang University, Nanchang, Jiangxi 330006, China; ^3^ Department of Animal Science, Medical College, Nanchang University, Nanchang, Jiangxi 330006, China; ^4^ Department of Experimental Teaching, Medical College, Nanchang University, Nanchang, Jiangxi 330006, China; ^5^ Jiangxi Province Key Laboratory of Tumor Pathogen's and Molecular Pathology, Nanchang, Jiangxi 330006, China

**Keywords:** miR-155-3p, myogenic enhance factor 2C, embryonic stem cell, cardiomyocyte, extracellular signal-regulate kinase

## Abstract

MicroRNAs (miRNAs) are short, noncoding RNAs that regulate post-transcriptional gene expression by targeting messenger RNAs (mRNAs) for cleavage or translational repression. Growing evidence indicates that miR-155 expression changes with the development of heart and plays an important role in heart physiopathology. However, the role of miR-155 in cardiac cells differentiation is unclear. Using the well-established embryonic stem cell (ESC), we demonstrated that miR-155-3p expression was down-regulated during cardiogenesis from mouse ESC. By contrast, the myogenic enhance factor 2C (MEF2C), a predicted target gene of miR-155-3p, was up-regulated. We further demonstrated that miR-155-3p inhibition increased the percentage of embryoid bodies (EB) beating and up-regulated the expression of cardiac specific markers, GATA4, Nkx2.5, and cTnT mRNA and protein. Notably, miR-155-3p inhibition caused upregulation of MEF2C, KRAS and ERK1/2. ERK1/2 inhibitor, PD98059 significantly decreased the expression of MEF2C protein. These findings indicate that miR-155-3p inhibition promotes cardiogenesis, and its mechanisms are involved in the RAS-ERK1/2 signaling and MEF2C.

## INTRODUCTION

Heart disease, including myocardial infarction, is the leading cause of death worldwide. Myocardial infarction causes irreversible damage to the myocardium due to the limited capacity of postnatal hearts to generate new cardiomyocytes. It is identified that cardiomyocytes lost by apoptosis are renewed at a rate of approximately 1% per year from birth [1]. Current medical therapies for heart disease can't replace the injury myocardium tissue with re-new cardiomyocytes.

Stem cell therapies have been investigated as a possible treatment approach for cardiac disease [2–4]. Although stem cell therapy strategies applied in patients have been extensively investigated in large animal studies [5–8], clinical success of cardiac cell-therapies remains limited [9]. Thus, studies on improving the efficiency of stem cell therapy need to be further investigated.

MicroRNA (miRNA) are a class of 21-25 nucleotides noncoding RNA that bind and inhibit the translation of target messenger RNAs. It is estimated that more than 30% of the human genome may be subjected to be regulated by miRNAs [10]. miRNAs are crucial regulators of cellular pathways in differentiation, development, apoptosis, metabolism and proliferation [11–13]. Growing evidence shows that miRNAs are involved in cardiogenesis and heart pathological process [14–16]. miR-590 and miR-199a are shown to promote cell cycle re-entry of adult cardiomyocytes *ex vivo* and enhance the cardiomyocyte proliferation. They stimulated marked cardiac regeneration and almost recovered cardiac functional parameters in a mouse myocardial infarction model [17]. MicroRNA also promoted cardiac progenitor cell proliferation and differentiation and facilitated the ESCs to differentiate into cardiomyocyte, which can potentially increase the efficiency stem cell therapy in cardiac diseases. For instance, over-expression of miR-17-92 increases the proliferation in adult cardiac progenitor cells *in vivo* by two-fold [18].

MiR-155 is located on chromosome 21 and transcribe from the B-cell integration cluster. It can be split into two mature microRNAs: miR-155-3p and miR-155-5p. MiR-155 is the most extensively investigated miRNA in immune cells [19–21]. Recently, it is also indentified that miR-155 plays a crucial roles in regulation cardiac disease. However the role of miR-155 cardiogenesis is still unknown.

In this study, we explored the expression levels of miR-155-3p during ESC differentiation into cardiomyocyte. The role of miR-155-3p inhibition in cardiogenesis *in vitro* and its potential mechanism were also investigated. Our novel understanding of the miR-155-3p inhibition in cardiogenesis could partly enlighten a new therapeutic strategy to heart disease.

## RESULTS

### miR-155-3p was down-regulated during ESCs differentiation

**Figure 1:**
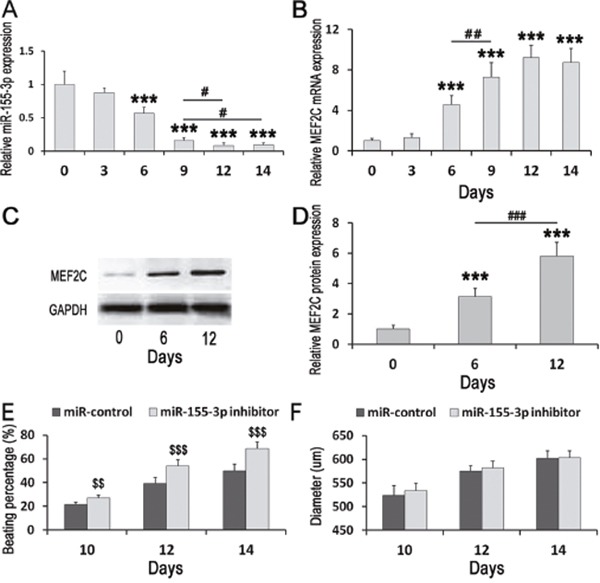
Expressions change of miR-155-3p and MEF2C during ESCs differentiation and effects of miR-155-3p inhibitor on EBs beating and growth **(A-C)** MEF2C mRNA and miR-155-3p were detected by Real-time PCR. MEF2C protein was measured by Western blot. (A) miR-155-3p level in the EBs at d0, d3, d6, d9, and d12 was detected. miR-155-3p was down-regulated during ESCs differentiation. (B) MEF2C mRNA in the EBs at d0, d3, d6, d9, and d12 was detected. MEF2C mRNA was up-regulated during ESCs differentiation. n=6. (C) MEF2C protein in the EBs at d0, d6, and d12 was measured. **(D)** Data indicated the expression level of MEF2C. MEF2C was significantly up-regulated in d6 group as compared to d0 group. n=5. **(E** and **F)** ESCs were stably transfected with miR-155-3p inhibitor. (E) miR-155-3p inhibitor promoted the EBs beating. The percentages of EBs beating were calculated at d10, d12, and d14. n=6. (F) miR-155-3p inhibitor did not change EBs growth. The diameters of EBs were measured at d10, d12, and d14. n=6. Data were showed as mean ± S.D. ^***^P < 0.001 *vs* d0 group; ^#^*P* < 0.05;^##^*P* < 0.01, and ^###^*P* < 0.001; ^$$^*P* < 0.01, and ^$$$^*P* < 0.001 vs control group.

The expression profile of miR-155-3p during ESCs differentiation was detected using real-time PCR. Analysis of miR-155-3p expression revealed that miR-155-3p was gradually down-regulated during cardiac differentiation of ESCs (Figure 1A). When compared to the d0 group, miR-155-3p level in the d3, d6, d9, and d12 groups was 0.875±0.0718, 0.565±0.0938, 0.155± 0.0469, and 0.083±0.0380 folds, respectively. At d14, miR-155-3p kept at low level and there had significant difference between d12 and d14 groups. MiR-155-3p mimic increased the expression of miR-155-3p and miR-155-3p mimic decreased the expression of miR-155-3p (Supplementary Figure 2).

### MEF2C was up-regulated during ESCs differentiation

The expression profiles of MEF2C mRNA and protein during ESCs differentiation were detected using real-time PCR and Western blot. Analysis of MEF2C mRNA expression revealed that MEF2C mRNA level was up-regulated since d6 of cardiac differentiation of ESCs (Figure 1B). When compared to the d0 group, MEF2C mRNA level in d3, d6, d9, d12, and d14 and groups was 1.31±0.36, 4.58±0.87, 7.29±1.41, and 9.23±1.23, and 8.75±1.37 folds, respectively. There was no significant difference until d6 as compared to the d0 group. MEF2C mRNA keep at high level at d14, but there had no significant difference between d9 and d14. When compared to d6 group, MEF2C mRNA in d9 group was significant higher. Analysis of MEF2C protein expression revealed that MEF2C was significantly up-regulated in d6 group as compared to d0 group. From d6 to d12, It continued to increase and the protein level in d12 group was 5.83±0.88 folds when compared to d0 group (Figures 1C and 1D).

### Effects of miR-155-3p inhibitor on EBs beating and growth

To evaluate the effect of miR-155-3p inhibition on EBs beating during cardiac differentiation of ESCs, the beating percentage of EBs was counted at d10, d12, and d14. The results indicated that miR-155-3p inhibitor significantly increased percentage of EBs beating (Figure 1E). At d10, the percentage of EBs beating in miR-155-3p inhibitor group (27.09%±2.25%) was significant increased as compared to control group (21.38%±2.03%). With the continuous differentiation, the percentage of EBs beating in both miR-155-3p inhibitor and control groups were increased when compared to the corresponding groups at d10. The percentage of EBs beating in miR-155-3p inhibitor group at d12 and d14 were 53.77%±5.16% (39.25%±4.99% in control) and 68.61%±5.58% (49.79% ±5.70% in control), respectively. Which were significantly increased as compared to the corresponding control groups. These results demonstrated that miR-155-3p inhibitor facilitated the EBs beating. However, as the results showed that miR-155-3p inhibitor did not change the diameters of EBs at d10, d12 and d14 (Figure 1F).

### miR-155-3p mimic inhibited the expression of MEF2C

**Figure 2:**
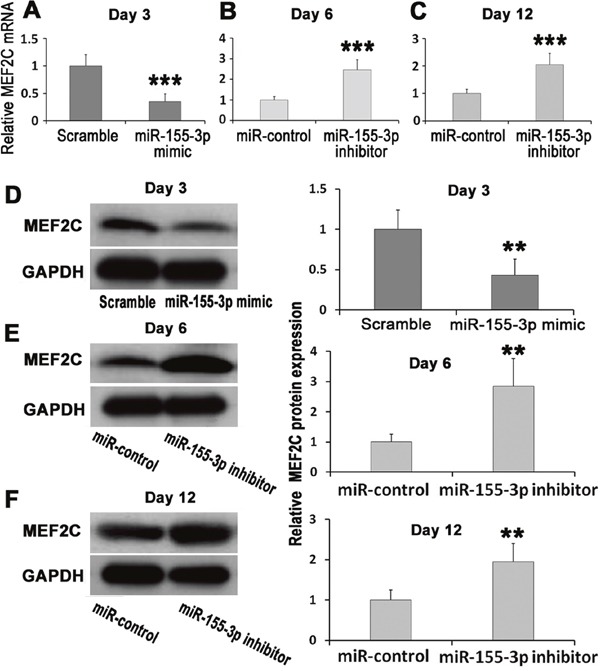
Effects of miR-155-3p mimic and miR-155-3p inhibition on expression of MEF2C ESCs were transiently transfected with miR-155-3p mimic or stably transfected with miR-155-3p inhibitor. **(A-C)** Expression change of MEF2C mRNA, n=6. (A) miR-155-3p mimic inhibited expression of MEF2C mRNA in EBs at d3. (B) miR-155-3p inhibitor increased expression of MEF2C mRNA in EBs at d6. (C) miR-155-3p inhibitor increased expression of MEF2C mRNA in EBs at d12. **(D-F)** Effects of miR-155-3p mimic and miR-155-3p inhibitor on expression of MEF2C protein, n=4. (D) miR-155-3p mimic inhibited expression of MEF2C protein in EBs at d3. (E) miR-155-3p inhibitor increased expression of MEF2C protein in EBs at d6. (F) miR-155-3p inhibitor increased expression of MEF2C protein in EBs at d12. Data were showed as mean ± S.D., n=6. ^**^P < 0.01, and ^***^P < 0.001 *vs* control or scramble group.

In order to investigate the effects of miR-155-3p mimic on the expression of MEF2C, ESCs were transiently transfected with miR-155-3p mimic. At d3 of EBs (3 days after transient transfection), MEF2C expression was detected using real-time PCR and Western blot. The results showed that miR-155-3p mimic suppressed both MEF2C mRNA (Figure 2A) and protein (Figure 2D) expressions. As compared to the corresponding scramble groups, the MEF2C mRNA and protein levels in miR-155-3p mimic groups were 0.35 ± 0.14 and 0.43±0.20 folds, respectively. These data suggested that miR-155-3p efficiently suppressed the expressions of MEF2C mRNA and protein.

### miR-155-3p inhibitor increased the expression of MEF2C during ESCs differentiation

To detect the effects of miR-155-3p inhibitor on expression of MEF2C, the ESCs were stablely transfected with miR-155-3p inhibitor. At d6 and d12, MEF2C expression was determined using real-time PCR and Western blot. The results showed that miR-155-3p inhibitor increased both MEF2C mRNA and protein expressions. When compared to the scramble groups, the MEF2C mRNA and protein levels in miR-155-3p inhibitor groups were 2.46±0.50 and 2.85±0.92 folds, respectively at d6 (Figure 2B and 2E). In addition, miR-155-3p inhibitor also increased the MEF2C mRNA (2.05±0.41 fold) and protein (1.95±0.43 fold) levels at d12 (Figure 2C and 2F). These results implied that miR-155-3p inhibitor promoted MEF2C expression might through inhibiting expression of miR-155-3p.

### miR-155-3p inhibitor promoted the expressions of cTnT, Nkx2.5 and gata4

**Figure 3:**
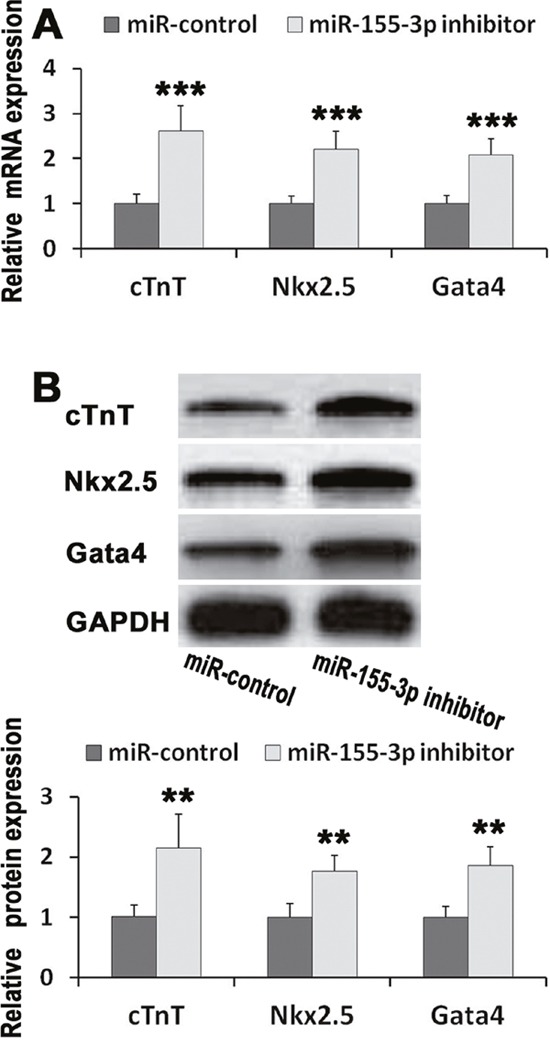
miR-155-3p inhibitor promoted expressions of cTnT, Nkx2. 5, and Gata4 mRNA was detected by real-time PCR. Protein levels were measured by western blot. cTnT, Nkx2.5, and Gata4 mRNA and protein were detected in EBs at d14. **(A)** miR-155-3p inhibitor increased expressions of cTnT, Nkx2.5, and Gata4 mRNA. n=6. **(B)** miR-155-3p inhibitor increased expressions of cTnT, Nkx2.5, and Gata4 protein. n=4. Data were showed as mean ± S.D., ^**^P < 0.01 and ^***^P < 0.001 *vs* control group.

To further investigate the effects of miR-155-3p inhibitor on cardiogenesis of ESCs, the expressions of cardiac specific markers, cTnT, Nkx2.5 and Gata4, were investigated at d14. Analysis of mRNA expression revealed that miR-155-3p inhibitor significantly improved the mRNA levels of cTnT, Nkx2.5 and Gata4 (Figure 3A). Similarly, we observed that miR-155-3p inhibitor significantly increased the expressions of cTnT, Nkx2.5 and Gata4 protein (Figure 3B). These findings indentified that miR-155-3p inhibitor promoted the cardiac differentiation of ESCs.

### miR-155-3p inhibitor increased the immunoreactivities of MEF2C, cTnT and Nkx2.5

**Figure 4:**
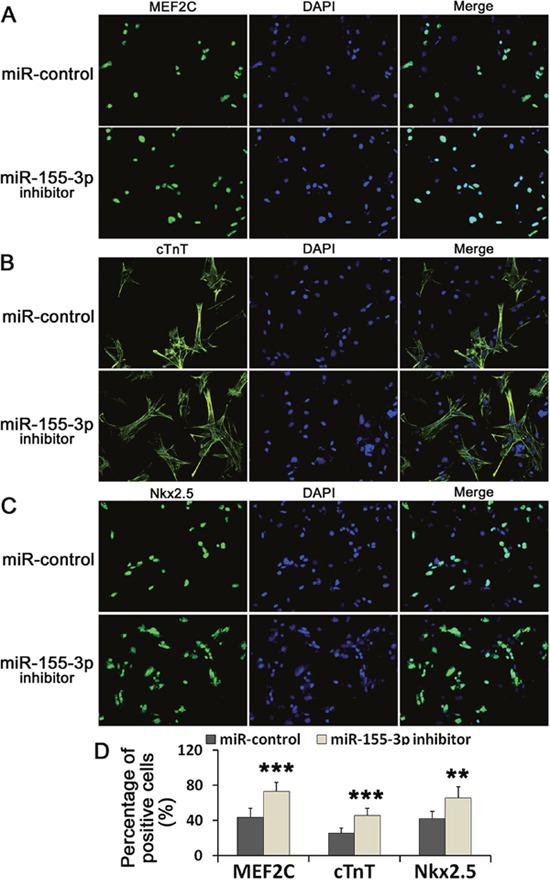
miR-155-3p inhibitor increased the immunoreactivity of MEF2C, cTnT and Nkx2.5 **(A-C)** The immunoreactivities of MEF2C, cTnT and Nkx2.5 were detected by immunofluorescence at d14. **(D)** Data showed the percentage of the positive cells. Data were showed as mean ± S.D., n=6. ^**^P < 0.01 and ^***^P < 0.001 *vs* control group.

To confirm the expression of cardiac specific markers, the immunoreactivities of MEF2C, cTnT and Nkx2.5 were detected using immunofluorescence at d14. The results showed that miR-155-3p inhibitor increased the percentage of MEF2C, cTnT and Nkx2.5 positive cells. The percentage of MEF2C, cTnT and Nkx2.5 positive cells in miR-155-3p inhibitor groups were 73.6%±10.2% (43.5%±10.2% in control), 45.6%±8.4% (25.9%±5.6% in control), and 66.0%±12.7% (42.0%±8.7% in control), respectively. When compared to the control group, the percentage of MEF2C, cTnT and Nkx2.5 positive cells in miR-155-3p inhibitor groups were significantly increased (Figure 4). These data further illustrated that miR-155-3p inhibition promoted the cardiac differentiation of ESCs.

### MEF2C was a direct target of miR-155-3p

**Figure 5:**
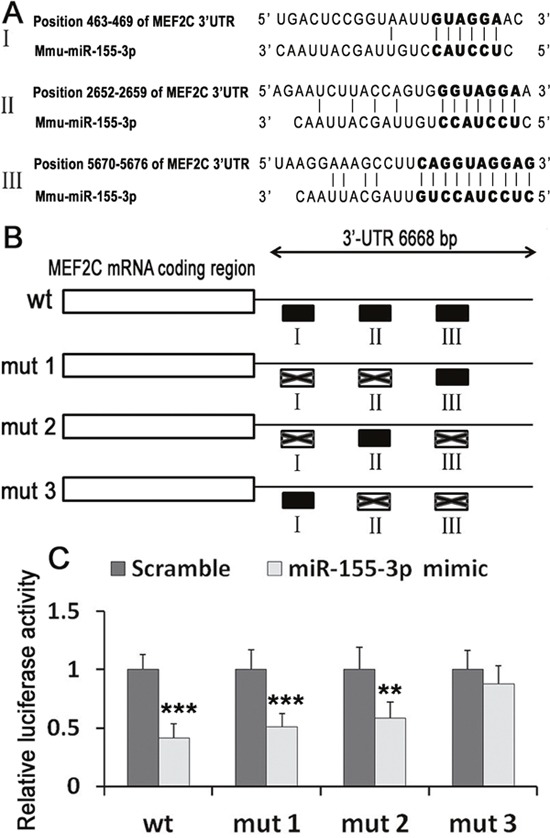
MEF2C was a direct target of miR-155-3p **(A)** Sequence alignment of miR-155-3p and its target sites in the 3’-UTR of MEF2C (download from http://www.targetscan.org/vert_71). **(B)** The seed regions of mutant MEF2C 3’-UTR were showed. **(C)** Luciferase activities were measured. Renill luciferase activities were normalized to firefly luciferase. Data were showed as mean ± S.D., n=6. ^**^P < 0.01 and ^***^P < 0.001 *vs* scramble group.

Sequence alignment of miR-155-3p and its target sites in the 3’-UTR of MEF2C were listed (Figure 5A). To test whether miR-155-3p could directly target the 3’-UTR of MEF2C mRNA in a sequence-specific manner, we generated a luciferase construct harbouring a potential binding site for miR-155-3p and produced three mutant constructs with potential target sites (Figure 5B). Without any mutation, miR-155-3p repressed 58.4% of luciferase activity of the reporter construct. Both mut 1(mutation sites of I and II) and mut 2 (mutation sites of I and III) repressed luciferase activity. However mut 3 (mutation sites of II and III) did not change luciferase activities (Figure 5C). The results suggested that miR-155-3p can repress MEF2C expression through the direct interaction with II or III.

### Effects of miR-155-3p on expressions of KRAS and pERK1/2

**Figure 6:**
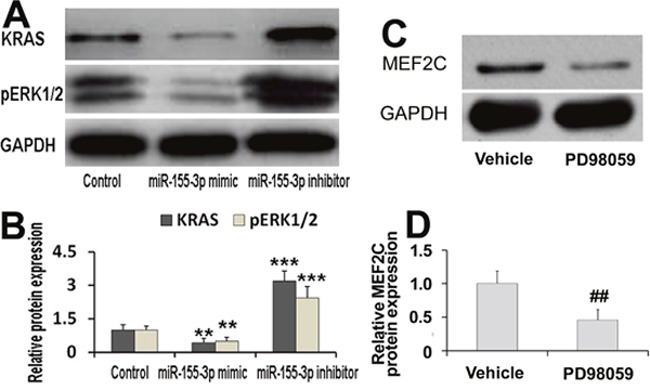
Effects of miR-155-3p on expressions of KRAS and pERK1/2 **(A, B)** KRAS and pERK1/2 protein levels were measured by Western blot. KRAS and pERK1/2 protein was detected in EBs at d3. miR-155-3p mimic repressed expressions of KRAS and pERK1/2. By contrast, miR-155-3p increased expressions of KRAS and pERK1/2. **(C, D)** ERK1/2 inhibitor, PD98059 significantly decreased expression of MEF2C protein. At d9, the EBs were treated with 50 μM PD98059 for 24 h. Data were showed as mean ± S.D., n=4. ^**^P < 0.01 and ^***^P < 0.001 *vs* control group; ^##^*P* < 0.01 *vs* vehicle group.

**Figure 7:**
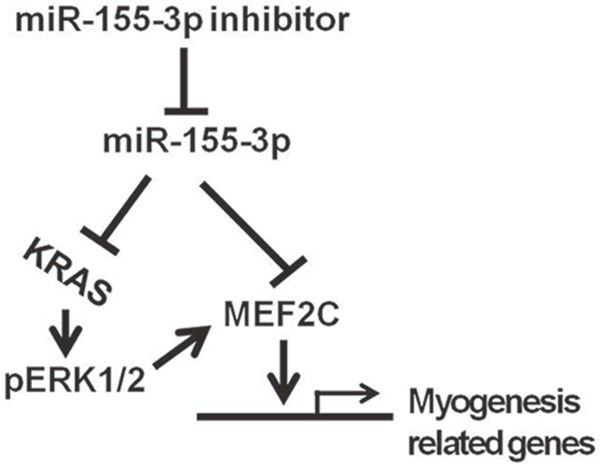
Schematic diagram showed the possible pathways of miR-155-3p inhibition-triggered cardiomyocyte differentiation of ESCs

To further investigate the mechanisms of miR-155-3p inhibition-triggered cardiomyocyte differentiation, the effects of miR-155-3p on protein levels of KRAS and pERK1/2 were evaluated by Western blot. The results showed that miR-155-3p mimic repressed expressions of KRAS and pERK1/2 in EBs at d3. By contrast, miR-155-3p inhibition promoted expressions of KRAS and pERK1/2 (Figure 6A, 6B). In addition, the effect of ERK1/2 inhibition on expression of MEF2C was explored. The results suggested that PD98059 significantly decreased expression of MEF2C protein (Figure 6C, 6D). Our data implied that KRAS/pERK1/2 was involved in the miR-155-3p inhibition-triggered cardiomyocyte differentiation. The possible pathways of KRAS/pERK1/2 signaling and MEF2C in miR-155-3p inhibition-triggered cardiomyocyte differentiation were showed in Figure 7.

## DISCUSSION

The present study demonstrated the following novel findings: (1) miR-155-3p was down-regulated during ESCs differentiation. (2) miR-155-3p mimic attenuated MEF2C expression in EBs. (3) miR-155-3p inhibition increased MEF2C, KRAS, and pERK1/2 expression. Its mechanisms may be involved in the inhibition of miR-155-3p. (4) miR-155-3p inhibition facilitated cardiogenesis of ESCs. These findings reveal the critical role of MEF2C, KRAS, and pERK1/2 in miR-155-3p inhibition-promoted cardiac differentiation.

The myocyte enhancer factor 2 (MEF2) proteins belong to the evolutionarily ancient MADS (MCM-1-agamous-deficiens-serum response factor) family of transcription factors. MEF2 family is detected in a wide range of tissues, but there are most abundant in striated muscles and brain [22, 23]. In mammals, this family has four members: MEF2A, MEF2B, MEF2C, MEF2D [24]. MEF2C is the first member to be expressed in the somite myotome, appearing initially in mesodermal precursors that give rise to the heart [23]. MEF2C regulates the expression of several cardiac structure and contractile proteins which are associated with the cardiogenesis [25].

Murine *Mef2c* gene is expressed in heart precursor cells before formation of the linear heart tube. In mice homozygous for a null mutation of *Mef2c*, the heart tube do not undergo looping morphogenesis, the future right ventricle do not form, and a subset of cardiac muscle genes is not expressed. *Mef2*-null mice exhibit cardiac looping defects and lead to lethality in homozygous mutants by embryonic day 9.5 [26]. A dominant-negative fusion protein of MEF2C with the engrailed repression (EnR) domain (MEF2C/EnR) in embryos results in a lack of heart structures and a severe disruption of cardiomyogenesis [27]. Similar to the findings *in vivo*, MEF2C plays crucial role in cardiomyogenesis *in vitro*. Over-expression of Gli2 up-regulates MEF2C mRNA and protein expression while enhancing cardiac differentiation of P19 ESC [28]. HH/GLI2 regulates the expression of Mef2c by recruiting BRG1 to the Mef2c gene, most probably via chromatin remodeling, to ultimately regulate *in vitro* cardiomyogenesis [29]. Fibroblasts can be directly reprogrammed into cardiomyocyte-like cells (iCMs) by over-expression of cardiac transcription factors, including Mef2c, Gata4, and Tbx5 [30]. Furthermore, DAPT, a classical notch inhibitor, enhances cardiac reprogramming by increasing binding of the transcription factor MEF2C to the promoter regions of cardiac structural genes [31]. Our previous data show that HDAC4 inhibition promotes MEF2C expression in the cardiac differentiation of ESCs [32]. In the current study, MEF2C expression was detected during cardiac differentiation of ESCs. The results showed that MEF2C was up-regulated during ESCs differentiation.

Growing evidence indicates that miR-155 is involved in the regulation of cardiac cell. miR-155 can reduce cardiac injury by inhibiting NF-kB pathway during acute viral myocarditis [33]. Systemic administration of miR-155 mimic attenuates cardiac dysfunction and improves late sepsis survival by targeting JNK associated inflammatory signaling [34]. However, inhibition of miR-155 using antagomiR improved cardiac function and suppressed cardiac apoptosis induced by lipopolysaccharide in mice. MiR-155 is also found to be up-regulated in the plasma of patients with septic cardiac dysfunction. Inhibition of miR-155 represents a novel therapy for septic myocardial dysfunction [35]. MicroRNA-155 expression is up-regulated and localized primarily in heart-infiltrating macrophages and CD4(+) T lymphocytes during acute myocarditis. microRNA-155 knockout mice develop attenuated viral myocarditis [36].

MiR-155-3p is down-regulated in heart of fetal and adult cardiac remodeling, an adaptive alteration that results an altered heart structure and function, when compared to control group. By contrast, miR-155-5p is up-regulated [37]. It has been shown that miR-155 level in proliferating cardiomyocyte progenitor cells is higher than differentiated cardiomyocyte progenitor [38]. However, there was no report regarding to the potential function of miR-155 in cardiac differentiation. In the present study, the expression profile of miR-155-3p during cardiac differentiation of ESCs was investigated. The results indicated that miR-155-3p was down-regulated during ESCs differentiation. Taking these data together, miR-155-3p seems to be involved in the regulation of cardiogenesis.

The target genes of miR-155-3p were investigated by using online bioinformatic tools targetScan (http://www.targetscan.org/vert_71/). We found that MEF2C was a predicted target gene of miR-155-3p. In this study, we confirmed that miR-155-3p suppressed MEF2C expression using Real-time PCR and Western blot. And luciferase results also showed that MEF2C was a direct target of miR-155-3p. Thus, miR-155-3p regulates cardiogenesis maybe through inhibiting MEF2C. In the present study, we indentified that miR-155-3p was expressed in ESCs and the expression level in ESCs was higher than in cardiocytes differentiated from ESCs. We supposed that miR-155-3p in the ESCs and early cardiac differentiation of ESCs would partly prevent the cardiogenesis through inhibition of MEF2C. Here, we focused on whether miR-155-3p inhibition could be used *in vitro* to induced cardiomyocyte differentiation. To further unveil the biological function of miR-155-3p inhibition in the process of cardiogenesis, the miR-155-3p inhibitor was stably transfected into ESCs and the cardiac differentiation was explored. The results suggested that miR-155-3p inhibition promoted the cardiomyocyte differentiation and increased MEF2C expression.

RAS proteins are small GTPases that play crucial roles in numerous functions such as control cell proliferation, differentiation and apoptosis. RAS has 3 isoforms, there are HRAS, KRAS, and NRAS. Activation of RAS trigger a broad range of downstream signaling pathways, including the RAF-MEK-Extracellular signal-regulate kinase (ERK) pathway [39]. In rat ventricular myocytes, activation of ERK1/2 is mediated through the classical Ras/Raf/MEK/ERK pathway, with Ras acting as the upstream trigger [40].

ERK is one of the three MAPKs (ERK, JNK, and p38). It had been identified that ERK was involved in the regulation of cardiac differentiation. Heregulin-beta1 promotes the development of cardiomyocytes derived from ES cell predominantly by activation of MEK-ERK [41]. In addition, sustained activation of ERK by 5-Azacytidine contributes to the induction of the differentiation of human mesenchymal stem cell into cardiomyocytes *in vitro* [42]. MAPKs participated in the regulation of MEF2C. MEF2A and MEF2C are preferentially phosphorylatied and activate [43]. ERK1/2-RSK2 signaling is a novel mechanism by which neurotrophins activates MEF2C and promotes neuronal survival [44]. In myocardial cell line H9C2, inhibition of ERK1/2 decreases alcohol-induced over-expression of GATA4 and MEF2C [45]. Our data suggested that ERK1/2 inhibitor decreased the expression of MEF2C protein. Thus, ERK1/2 triggering cardiac differentiation may be related to the regulation of MEF2C.

It was well known that MAPKs pathway could be regulated by microRNAs. In chronic myeloid leukemia cells, miR-155 regulates MAPKs through targeting KRAS or SOS, upstream signaling of MAPKs [46]. Reduced miR-155, miR-134, miR-373, miR-138, miR-205, miR-181d, miR-181c, and let-7 in CAsE-PE cells correlate with increased KRAS protein [47]. Furthermore, miR-155 can decrease the expressions of KRAS protein and mRNA [48]. Interestingly, miR-155 is the identified miRNA which regulates nucleus pulpous cells degeneration through directly targeting ERK1/2 [49]. Our data showed that miR-155-3p inhibited KRAS and pERK1/2 expression in EBs. By contrast, miR-155-3p inhibition increased KRAS and pERK1/2 expression. Thus, KRAS/ERK1/2/MEF2C pathway may be involved in the miR-155-3p inhibition-facilitated cardiomyocyte differentiation from ESCs.

In conclusion, our data indicated that miR-155-3p was down-regulated during cardiac differentiation of ESCs. miR-155-3p inhibited the expression of MEF2C. miR-155-3p inhibition increased the percentage of EBs beating and facilitated the expressions of MEF2C, GATA4, Nkx2.5, and cTnT. In addition, miR-155-3p inhibition increased KRAS and pERK1/2 expression. These data illustrated that miR-155-3p inhibition promoted cardiac differentiation of ESCs and its mechanisms are involved in RAS-ERK1/2 signaling and MEF2C.

## EXPERIMENTAL PROCEDURES

### Mouse ESCs culture

Mouse ESCs (CGR8; ECACC, Porton Down Salisbury UK) were cultivated in gelatin-coated petri-dishes in ESC culture medium consisting of DMEM (Gibco), 1-non-essential amino acids (Gibco), 2 mmol/L L-glutamine (Gibco), 0.1 mmol/L β-mercaptoethanol (Gibco), 1 mmol/L sodium pyruvate (Sigma), 100 U/ml penicillin (Gibco), 100 μg/ml streptomycin (Gibco), 15% fetal bovine serum (Hyclone), and 10^3^ U/ml LIF (Leukemia inhibitory factor; Chemicon). Cells were maintained in a of 5% CO2 atmosphere at 37°C.

### miRNA transfection

Mmu-miR-155-3p Inhibitor (Invitrogen, 100 nM) and miRNA inhibitors-negative control (Invitrogen, 100 nM) were cloned into the pcDNA3.1 vector to yield pcDNA-miR-155-3p Inhibitor and pcDNA-control, respectively.

CGR8 ESCs (2×10^5^/well) were plated into 6-well plates and grown for one day in ESCs culture medium. The cells at 60% confluence were transfected with mmu-miR-155-3p mimic (Invitrogen, 50 nM), negative scramble (Invitrogen, 50 nM), pcDNA-miR-155-3p inhibitor or pcDNA-control in serum-free medium by Lipofectamine 2000 (Invitrogen, USA) according to the manufacturer's instructions. Transfected cells were incubated in ESC culture medium. The stable clones of pcDNA-miR-155-3p inhibitor and pcDNA-control were generated by growing cells in the presence of G418 at 500 μg/ml for 2 weeks.

### *In vitro* differentiation

CGR8 ESCs transfected with miR-155-3p inhibitor or control were dissociated by 0.25% trypsin-0.01% EDTA and suspended in the differentiation medium (ESCs culture medium without LIF). The cell drops (400 cells/20 μl) were placed onto the lids of culture dishes. The lips were reverted and then cover the dishes which contain 2 ml deionized water. After 2 days EB formation by the hanging drop method, Embryoid bodies (EBs) were transferred to non-coated petri dishes for suspension culture up to day 7 (d7). For diameter measurement, EBs were further cultured in non-coated culture dishes. For differentiation assay, EBs were cultured in gelatin-coated 96-well culture plates (1 EB/well). The EBs growth were monitored at day 10 (d10), d12 and d14. The spontaneous rhythmic contraction of EBs was analyzed at d10, d12 and d14. To test the effects of ERK1/2 on the regulation of MEF2C, EBs at d9 were treated with ERK1/2 inhibitor, PD98059 (50 μM) for 24 h. The differentiation experimental protocol was showed in Supplementary Figure 1.

### Real-time PCR quantification

Total RNA was extracted using TRIzol (Invitrogen, Carlsbad, CA, USA) following the manufacturer's protocol. 1000 ng total RNA was used as a template for reverse transcription. For protein-coding mRNA, cDNA was obtained with Applied Biosystems Reverse Transcription Kit (Applied Biosystems, Foster City, CA, USA). For miR-155-3p, cDNA was obtained with TaqMan® MicroRNA Reverse Transcription Kit (Applied Biosystems, Foster City, CA, USA). Real-time quantitative PCR was performed using ABI7500 real-time PCR system (Applied Biosystems, Foster City, CA, USA). The expression levels of miR-155-3p were normalized to U6, while levels of MEF2C, cTnT, nkx2.5, and GATA4 were normalized to GAPDH. The miR-155-3p specific primers and MEF2C, cTnT, nkx2.5, and GATA4 probes were purchased from Applied Biosystems.

### Luciferase assay

Full length of 3’UTR of MEF2C were inserted into the pmirGLO vector (Promega, Madison, WI, USA) downstream of the renilla luciferase reporter gene (MEF2C-3’UTR-wt). The MEF2C-3’UTR-mut (mut 1, mut 2 and mut 3) were generated by point mutation of MEF2C-3’URT-wt based on the predicted target sites. ESCs were co-transfected with the reporter vectors containing the MEF2C-3’UTR-wt or MEF2C-3’UTR-mut and miR-155-3p mimic or scramble by using lipofectamine 2000 (Invitrogen, Carlsbad, CA, USA). The cells were harvested 48 h later, and luciferase activity was assessed using a Dual Luciferase Reporter Assay System (Promega). Firefly luciferase was used to normalise the renilla luciferase.

### Western blot

The cells were lysed with RIPA buffer (150mM sodium chloride, 1.0% NP-40, 0.5% sodium deoxycholate, 0.1% SDS, 50mM Tris, pH 8.0), supplementing with protease inhibitor cocktail. Protein concentrations were determined using Bradford protein assay reagent with bovine serum albumin (BSA) as a standard. The protein lysate (30 μg) was subjected to electrophoresis on 10 % (w/v) sodium dodecyl sulphate–polyacrylamide gel electrophoresis (SDS–PAGE) and transferred to polyvinlylidene difluoride (PVDF) membranes (Bio-Rad Laboratories, Hercules, CA, USA). The membrane was blocked with 5% (w/v) BSA in Tris-buffered saline (TBS, pH 7.6) for 2 h at room temperature. After incubation with primary antibody of MEF2C (1:1000; Abcam), KRAS (1:1000; Abcam), pERK1/2 (1:1000; Santa Cruz), cTnT (1:500; Abcam), GATA4 (1:500; Abcam), or Nkx2.5 (1:500; Santa cruze) overnight at 4°C, the membrane was then incubated with peroxidase conjugated secondary antibodies (1:5000; Cell SignalingTechnology) for 1h at room temperature. Immunodetection was completed using Pierce-enhanced chemiluminescence substrate (Thermo Scientific, USA) and then exposed to X-ray film. GAPDH (1:10000; Abcam) was used as a loading control.

### Immunofluorescence

The differentiated ESCs (d14) were digested and separated into single cell. After washing with differentiation culture medium for 3 times, the cells were plated into coated coverslips for 12 hours. The coverlips were rinsed with PBS for 3 times and fixed in 4% paraformaldehyde in PBS for 30 minutes and permeabilized with 0.1% Triton X-100 in PBS for 5 min. The cells were incubated overnight at 4°C with anti-GATA4 (1:200), anti-MEF2C (1:300), anti-cTnT (1:300) or anti-Nkx2.5 (1:200) antibody. Subsequently the cells were incubated FITC- or Cy3- conjugated secondary antibody (1:200) for 1 hour at room temperature. The nucleis were stained with 4’, 6-diamidino-2-phenylindole (DAPI). The numbers of cTnT, GATA4, MEF2C or Nkx2.5 positive cells on each coverslip were counted in four randomized fields. The cells were observed at 20 times of the microscope. In each field, there were 50-100 cells. The positive cells were identified by the same person. The measurements were done under blind conditions.

### Statistical analysis

Data are showed as the mean ± S.D. Comparisons of means between two groups were carried out using a *t*-test. Statistical comparisons were performed by analysis of variance (ANOVA) with Dunnett's test for multiple comparisons. A value of *P* < 0.05 was considered to be significant.

## SUPPLEMENTARY MATERIALS FIGURES AND TABLES



## References

[1] Bergmann O, Bhardwaj RD, Bernard S, Zdunek S, Barnabe-Heider F, Walsh S, Zupicich J, Alkass K, Buchholz BA, Druid H, Jovinge S, Frisen J (2009). Evidence for cardiomyocyte renewal in humans. Science.

[2] Cambria E, Steiger J, Gunter J, Bopp A, Wolint P, Hoerstrup SP, Emmert MY (2016). Cardiac Regenerative Medicine: The Potential of a New Generation of Stem Cells. Transfus Med Hemother.

[3] Cho SJ, Kim SY, Jeong HC, Cheong H, Kim D, Park SJ, Choi JJ, Kim H, Chung HM, Moon SH, Cha HJ (2015). Repair of Ischemic Injury by Pluripotent Stem Cell Based Cell Therapy without Teratoma through Selective Photosensitivity. Stem Cell Reports.

[4] Emmert MY, Wolint P, Jakab A, Sheehy SP, Pasqualini FS, Nguyen TD, Hilbe M, Seifert B, Weber B, Brokopp CE, Macejovska D, Caliskan E, von Eckardstein A (2017). Safety and efficacy of cardiopoietic stem cells in the treatment of post-infarction left-ventricular dysfunction-From cardioprotection to functional repair in a translational pig infarction model. Biomaterials.

[5] Ban K, Park HJ, Kim S, Andukuri A, Cho KW, Hwang JW, Cha HJ, Kim SY, Kim WS, Jun HW, Yoon YS (2014). Cell therapy with embryonic stem cell-derived cardiomyocytes encapsulated in injectable nanomatrix gel enhances cell engraftment and promotes cardiac repair. Acs Nano.

[6] Chong JJ, Yang X, Don CW, Minami E, Liu YW, Weyers JJ, Mahoney WM, Van Biber B, Cook SM, Palpant NJ, Gantz JA, Fugate JA, Muskheli V (2014). Human embryonic-stem-cell-derived cardiomyocytes regenerate non-human primate hearts. Nature.

[7] Freund C, Mummery CL (2009). Prospects for pluripotent stem cell-derived cardiomyocytes in cardiac cell therapy and as disease models. J Cell Biochem.

[8] Menasche P, Vanneaux V, Fabreguettes JR, Bel A, Tosca L, Garcia S, Bellamy V, Farouz Y, Pouly J, Damour O, Perier MC, Desnos M, Hagege A (2015). Towards a clinical use of human embryonic stem cell-derived cardiac progenitors: a translational experience. Eur Heart J.

[9] Chen CH, Sereti KI, Wu BM, Ardehali R (2015). Translational aspects of cardiac cell therapy. J Cell Mol Med.

[10] Bartel DP (2009). MicroRNAs: target recognition and regulatory functions. Cell.

[11] Ambros V (2004). The functions of animal microRNAs. Nature.

[12] Da CMP, Leptidis S, Salic K, De Windt LJ (2010). MicroRNA regulation in cardiovascular disease. Curr Drug Targets.

[13] Gao Y, Ma X, Yao Y, Li H, Fan Y, Zhang Y, Zhao C, Wang L, Ma M, Lei Z, Zhang X (2016). miR-155 regulates the proliferation and invasion of clear cell renal cell carcinoma cells by targeting E2F2. Oncotarget.

[14] Liu N, Olson EN (2010). MicroRNA regulatory networks in cardiovascular development. Dev Cell.

[15] Zhu J, Yao K, Wang Q, Guo J, Shi H, Ma L, Liu H, Gao W, Zou Y, Ge J (2016). Ischemic Postconditioning-Regulated miR-499 Protects the Rat Heart Against Ischemia/Reperfusion Injury by Inhibiting Apoptosis through PDCD4. Cell Physiol Biochem.

[16] Cui Y, Li T, Yang D, Li S, Le W (2016). miR-29 regulates Tet1 expression and contributes to early differentiation of mouse ESCs. Oncotarget.

[17] Eulalio A, Mano M, Dal Ferro M, Zentilin L, Sinagra G, Zacchigna S, Giacca M (2012). Functional screening identifies miRNAs inducing cardiac regeneration. Nature.

[18] Sirish P, Lopez JE, Li N, Wong A, Timofeyev V, Young JN, Majdi M, Li RA, Chen HS, Chiamvimonvat N (2012). MicroRNA profiling predicts a variance in the proliferative potential of cardiac progenitor cells derived from neonatal and adult murine hearts. J Mol Cell Cardiol.

[19] Na SY, Park MJ, Park S, Lee ES (2016). MicroRNA-155 regulates the Th17 immune response by targeting Ets-1 in Behcet’s disease. Clin Exp Rheumatol.

[20] Rothchild AC, Sissons JR, Shafiani S, Plaisier C, Min D, Mai D, Gilchrist M, Peschon J, Larson RP, Bergthaler A, Baliga NS, Urdahl KB, Aderem A (2016). MiR-155-regulated molecular network orchestrates cell fate in the innate and adaptive immune response to Mycobacterium tuberculosis. Proc Natl Acad Sci USA.

[21] Slezak-Prochazka I, Kluiver J, de Jong D, Smigielska-Czepiel K, Kortman G, Winkle M, Rutgers B, Koerts J, Visser L, Diepstra A, Kroesen BJ, van den Berg A (2016). Inhibition of the miR-155 target NIAM phenocopies the growth promoting effect of miR-155 in B-cell lymphoma. Oncotarget.

[22] Dietrich JB (2013). The MEF2 family and the brain: from molecules to memory. Cell Tissue Res.

[23] Edmondson DG, Lyons GE, Martin JF, Olson EN (1994). Mef2 gene expression marks the cardiac and skeletal muscle lineages during mouse embryogenesis. Development.

[24] Potthoff MJ, Olson EN (2007). MEF2: a central regulator of diverse developmental programs. Development.

[25] Olson EN (2006). Gene regulatory networks in the evolution and development of the heart. Science.

[26] Lin Q, Schwarz J, Bucana C, Olson EN (1997). Control of mouse cardiac morphogenesis and myogenesis by transcription factor MEF2C. Science.

[27] Karamboulas C, Dakubo GD, Liu J, De Repentigny Y, Yutzey K, Wallace VA, Kothary R, Skerjanc IS (2006). Disruption of MEF2 activity in cardiomyoblasts inhibits cardiomyogenesis. J Cell Sci.

[28] Voronova A, Al Madhoun A, Fischer A, Shelton M, Karamboulas C, Skerjanc IS (2012). Gli2 and MEF2C activate each other’s expression and function synergistically during cardiomyogenesis in vitro. Nucleic Acids Res.

[29] Fair JV, Voronova A, Bosiljcic N, Rajgara R, Blais A, Skerjanc IS (2016). BRG1 interacts with GLI2 and binds Mef2c gene in a hedgehog signalling dependent manner during in vitro cardiomyogenesis. BMC Dev Biol.

[30] Yamakawa H, Muraoka N, Miyamoto K, Sadahiro T, Isomi M, Haginiwa S, Kojima H, Umei T, Akiyama M, Kuishi Y, Kurokawa J, Furukawa T, Fukuda K, Ieda M (2015). Fibroblast Growth Factors and Vascular Endothelial Growth Factor Promote Cardiac Reprogramming under Defined Conditions. Stem Cell Reports.

[31] Abad M, Hashimoto H, Zhou H, Morales MG, Chen B, Bassel-Duby R, Olson EN (2017). Notch Inhibition Enhances Cardiac Reprogramming by Increasing MEF2C Transcriptional Activity. Stem Cell Reports.

[32] Chen HP, Denicola M, Qin X, Zhao Y, Zhang L, Long XL, Zhuang S, Liu PY, Zhao TC (2011). HDAC inhibition promotes cardiogenesis and the survival of embryonic stem cells through proteasome-dependent pathway. J Cell Biochem.

[33] Bao JL, Lin L (2014). MiR-155 and miR-148a reduce cardiac injury by inhibiting NF-kappaB pathway during acute viral myocarditis. Eur Rev Med Pharmacol Sci.

[34] Zhou Y, Song Y, Shaikh Z, Li H, Zhang H, Caudle Y, Zheng S, Yan H, Hu D, Stuart C, Yin D (2017). MicroRNA-155 attenuates late sepsis-induced cardiac dysfunction through JNK and beta-arrestin 2. Oncotarget.

[35] Wang H, Bei Y, Huang P, Zhou Q, Shi J, Sun Q, Zhong J, Li X, Kong X, Xiao J (2016). Inhibition of miR-155 protects against LPS-induced cardiac dysfunction and apoptosis in mice. Mol Ther Nucleic Acids.

[36] Zhang Y, Zhang M, Li X, Tang Z, Wang X, Zhong M, Suo Q, Zhang Y, Lv K (2016). Silencing microRNA-155 attenuates cardiac injury and dysfunction in viral myocarditis via promotion of M2 phenotype polarization of macrophages. Sci Rep.

[37] Yan H, Li Y, Wang C, Zhang Y, Liu C, Zhou K, Hua Y (2017). Contrary microRNA Expression Pattern Between Fetal and Adult Cardiac Remodeling: Therapeutic Value for Heart Failure. Cardiovasc Toxicol.

[38] Sluijter JP, van Mil A, van Vliet P, Metz CH, Liu J, Doevendans PA, Goumans MJ (2010). MicroRNA-1 and -499 regulate differentiation and proliferation in human-derived cardiomyocyte progenitor cells. Arterioscler Thromb Vasc Biol.

[39] Moodie SA, Willumsen BM, Weber MJ, Wolfman A (1993). Complexes of Ras. GTP with Raf-1 and mitogen-activated protein kinase kinase. Science.

[40] Haworth RS, Dashnyam S, Avkiran M (2006). Ras triggers acidosis-induced activation of the extracellular-signal-regulated kinase pathway in cardiac myocytes. Biochem J.

[41] Kim HS, Cho JW, Hidaka K, Morisaki T (2007). Activation of MEK-ERK by heregulin-beta1 promotes the development of cardiomyocytes derived from ES cells. Biochem Biophys Res Commun.

[42] Qian Q, Qian H, Zhang X, Zhu W, Yan Y, Ye S, Peng X, Li W, Xu Z, Sun L, Xu W (2012). 5-Azacytidine induces cardiac differentiation of human umbilical cord-derived mesenchymal stem cells by activating extracellular regulated kinase. Stem Cells Dev.

[43] Yang SH, Galanis A, Sharrocks AD (1999). Targeting of p38 mitogen-activated protein kinases to MEF2 transcription factors. Mol Cell Biol.

[44] Wang Y, Liu L, Xia Z (2007). Brain-derived neurotrophic factor stimulates the transcriptional and neuroprotective activity of myocyte-enhancer factor 2C through an ERK1/2-RSK2 signaling cascade. J Neurochem.

[45] Gao W, Pan B, Liu L, Huang X, Liu Z, Tian J (2015). Alcohol exposure increases the expression of cardiac transcription factors through ERK1/2-mediated histone3 hyperacetylation in H9c2 cells. Biochem Biophys Res Commun.

[46] Chakraborty C, Sharma AR, Patra BC, Bhattacharya M, Sharma G, Lee SS (2016). MicroRNAs mediated regulation of MAPK signaling pathways in chronic myeloid leukemia. Oncotarget.

[47] Ngalame NN, Tokar EJ, Person RJ, Xu Y, Waalkes MP Aberrant microRNA expression likely controls RAS oncogene activation during malignant transformation of human prostate epithelial and stem cells by arsenic. Toxicol Sci.

[48] Forzati F, De Martino M, Esposito F, Sepe R, Pellecchia S, Malapelle U, Pellino G, Arra C, Fusco A (2017). miR-155 is positively regulated by CBX7 in mouse embryonic fibroblasts and colon carcinomas, and targets the KRAS oncogene. BMC Cancer.

[49] Ye D, Dai L, Yao Y, Qin S, Xie H, Wang W, Liang W (2016). miR-155 Inhibits Nucleus Pulposus Cells’ Degeneration through Targeting ERK 1/2. Dis Markers.

