# Supplementary material for: Involment of RAS/ERK1/2 signaling and MEF2C in miR-155-3p inhibition-triggered cardiomyocyte differentiation of embryonic stem cell
Source: Oncotarget. 2017 Sep 23;8(48):84403–16. doi: 10.18632/oncotarget.21218 (PMC5663606; doi:10.18632/oncotarget.21218)
Supplement: Supplementary file 1 [file oncotarget-08-84403-s001.pdf]

Involment of RAS/ERK1/2 signaling and MEF2C in miR-155-3p inhibition-triggered cardiomyocyte differentiation of embryonic stem cell

SUPPLEMENTARY MATERIALS

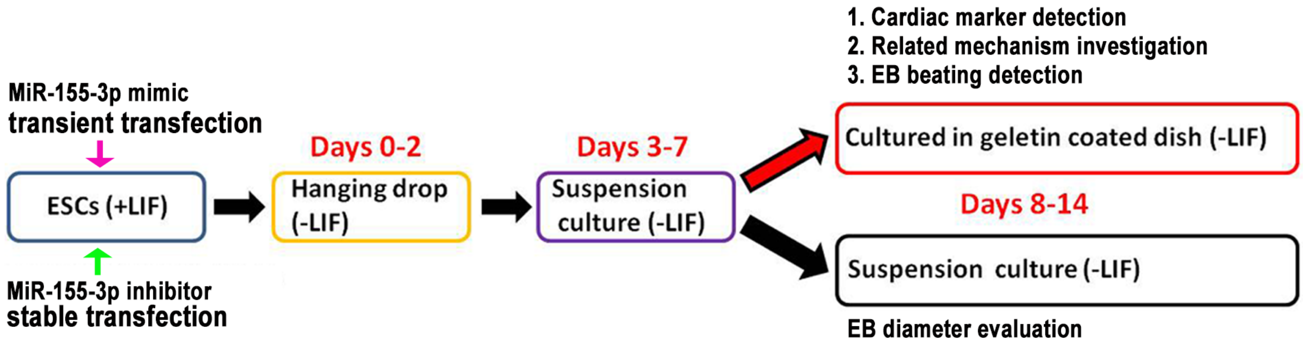

Supplementary Figure 1: The differentiation experimental protocol.

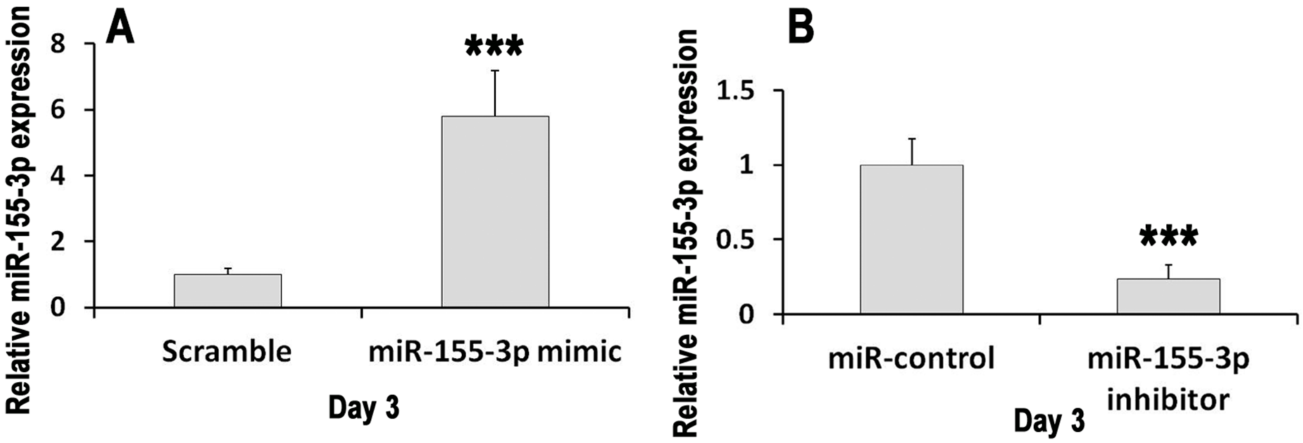

Supplementary Figure 2: Effects of miR-155-3p mimic and inhibitor on the expression of miR-155-3p. **(A)** MiR-155-3p mimic increased the expression of miR-155-3p. **(B)** MiR-155-3p mimic decreased the expression of miR-155-3p. Data were showed as mean ± S.D., n=6. $^{***}P < 0.001$ *vs* scramble group.
